# Petrosal ganglion: a more complex role than originally imagined

**DOI:** 10.3389/fphys.2014.00474

**Published:** 2014-12-04

**Authors:** Mauricio A. Retamal, Edison P. Reyes, Julio Alcayaga

**Affiliations:** ^1^Facultad de Medicina, Centro de Fisiología Celular e Integrativa, Clínica Alemana Universidad del DesarrolloSantiago, Chile; ^2^Dirección de Investigación, Universidad Autónoma de ChileTemuco, Chile; ^3^Laboratorio de Fisiología Celular, Departamento de Biología, Facultad de Ciencias, Universidad de ChileSantiago, Chile

**Keywords:** petrosal ganglia, sensory modality, chemosensory, mechanosensory, action potential

## Abstract

The petrosal ganglion (PG) is a peripheral sensory ganglion, composed of pseudomonopolar sensory neurons that innervate the posterior third of the tongue and the carotid sinus and body. According to their electrical properties PG neurons can be ascribed to one of two categories: (i) neurons with action potentials presenting an inflection (hump) on its repolarizing phase and (ii) neurons with fast and brisk action potentials. Although there is some correlation between the electrophysiological properties and the sensory modality of the neurons in some species, no general pattern can be easily recognized. On the other hand, petrosal neurons projecting to the carotid body are activated by several transmitters, with acetylcholine and ATP being the most conspicuous in most species. Petrosal neurons are completely surrounded by a multi-cellular sheet of glial (satellite) cells that prevents the formation of chemical or electrical synapses between neurons. Thus, PG neurons are regarded as mere wires that communicate the periphery (i.e., carotid body) and the central nervous system. However, it has been shown that in other sensory ganglia satellite glial cells and their neighboring neurons can interact, partly by the release of chemical neuro-glio transmitters. This intercellular communication can potentially modulate the excitatory status of sensory neurons and thus the afferent discharge. In this mini review, we will briefly summarize the general properties of PG neurons and the current knowledge about the glial-neuron communication in sensory neurons and how this phenomenon could be important in the chemical sensory processing generated in the carotid body.

## Introduction

The petrosal ganglion (PG) contains the soma of pseudomonopolar sensory neurons (Ramón y Cajal, 1909) that project to the posterior third of the tongue and the carotid sinus (CS) and body (CB) (Stensaas and Fidone, 1977). Although morphologically similar they constitute a heterogeneous population with regard to their sensory modality: both mechano–and chemosensory neurons can be recognized projecting to the periphery. Although there is no complete characterization of the population of neurons that project to the tongue, the neurons projecting to the carotid bifurcation appear to be segregated in terms of their electrophysiological (Belmonte and Gallego, 1983; Cummins et al., 2002; Varas et al., 2003) and morphological (Katz et al., 1983; Katz and Black, 1986; Kummer and Habeck, 1992) characteristics, the receptors that are expressed in their soma and the neurotransmitter that activate them (González et al., 1994; Iturriaga and Alcayaga, 2004; Nurse and Piskuric, 2013). As mentioned, PG neurons express receptors in their plasma membrane and respond to exogenous neurotransmitters application, characteristics that are present in other sensory ganglia in which there is intra-ganglionic information processing. Thus, we propose that PG have all the necessary components for intra-ganglionic information processing, and this may represent a future line of study in the carotid body-cardiorespiratory control.

## Electrophysiological properties of PG neurons

**Figure 1:**
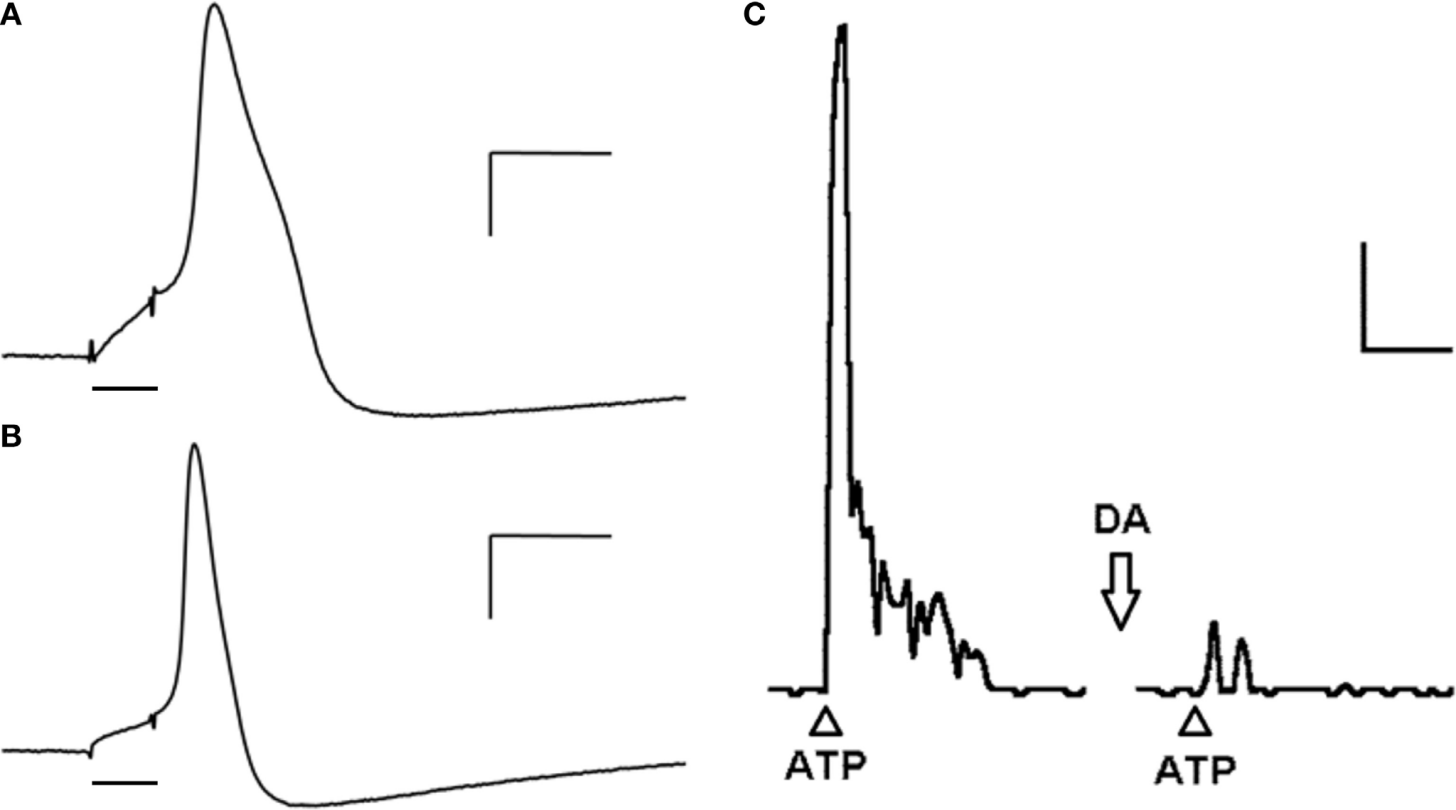
**Cat petrosal ganglion (PG) neurons in culture action potentials (APs), evoked by depolarizing pulses (bar), present an inflection (hump) in the repolarizing phase and long lasting hyperpolarization (A) or a smooth repolarizing phase and short hyperpolarization (B)**. Calibration: 10 mV, 5 ms. **(C)** A single dose of ATP (500 μg; arrowhead) applied to the PG increases transiently the frequency of discharge recorded from the carotid nerve. When the same dose of ATP is applied (arrowhead) after 30 s of a dose of dopamine (arrow; 1 mg) the response is significantly decreased. Calibration: 20 Hz, 15 s. Figure modified from Alcayaga et al. (2003a,b).

Intracellular recordings show two major populations of neurons according to their action potential (AP) waveform: neurons with APs presenting an inflection (hump) on its repolarizing phase (Figure 1A) and neurons with fast and brisk APs (Figure 1B) (Belmonte and Gallego, 1983; Morales et al., 1987; Varas et al., 2003). Whole cell recordings from cultured PG neurons of the rat nodose-petrosal-jugular complex (NPJc) indicate that all neurons present Na^+^ inward currents, although in 50% of these cells are tetrodotoxin (TTX)-resistant (Stea and Nurse, 1992). Ca^2+^ inward currents are also present in all neurons, mostly L-type. Outward K^+^ currents are comprised by both the delayed rectifier and Ca^2+^-dependent K^+^ currents, the latter representing about 20% of the total outward current (Stea and Nurse, 1992). Most neurons (76%) respond with a single AP to long depolarizing pulses while the remaining ones responds with two or more APs to the same stimuli (Stea and Nurse, 1992). Similarly, intracellular recordings from cat PG neurons indicate that neurons with humped APs present both TTX-sensitive and insensitive components (Gallego, 1983; Iturriaga et al., 2007), and that the depolarizing phase of the AP has an important Ca^2+^ component (Gallego, 1983). Reduction or blockade of Ca^2+^ currents reduces the AP amplitude and also the duration and amplitude of the AP hyperpolarization, suggesting the involvement of Ca^2+^-dependent K^+^ currents in the latter response (Gallego, 1983). On the other hand, the spikes of neurons with brisk APs are completely blocked by TTX (Belmonte and Gallego, 1983; Iturriaga et al., 2007). Because many of these recordings were performed on cultured or isolated PG neurons, there is no information on the sensory modality or peripheral target of the recorded neurons.

The central axotomy has no significant effect on PG neurons that present a hump in the AP, irrespective of their peripheral projection. However, peripheral axotomy decreases the conduction velocity, after hyperpolarization amplitude but increases AP duration without modifying resting membrane potential (Vm) or input resistance (Rin) (Belmonte et al., 1988). After axotomy spiking characteristics remained unchanged in neurons projecting through the glossopharyngeal branch (GPB), but the tonically discharging neurons projecting through the CSN increased by a factor of four (Gallego et al., 1987; Belmonte et al., 1988). Thus, electrical properties appear to be differentially modified according to the peripheral targets.

## Petrosal ganglion neurons projecting through the carotid sinus nerve

### Electrophysiological properties

Cat PG neurons projecting through the CSN with myelinated axons can also be categorized according to their AP waveform. Thus, sensory neurons connected to the CB present humped APs with longer hyperpolarizations and phasic responses (Belmonte and Gallego, 1983; Varas et al., 2003). Conversely, barosensory neurons projecting to the CS present brisk APs with shorter hyperpolarizations and tonic discharges to long lasting depolarizations (Belmonte and Gallego, 1983; Varas et al., 2003), although humped APs have also been recorded in barosensory neurons (Belmonte and Gallego, 1983). In the cat, neurons with unmyelinated axons (C-fiber) have similar characteristics to myelinated ones (Varas et al., 2003), although tonic responses have also been reported in few of them (Belmonte and Gallego, 1983). Thus, cat PG neurons projecting through the CSN can be set apart by their AP waveform and spiking characteristics, which are highly correlated with the sensory modality they convey to the central nervous system (Varas et al., 2003). On the other hand, mouse and rat chemosensory PG neurons have unmyelinated axons (Donnelly, 1999; Donnelly and Rigual, 2000), with rat neurons presenting humped APs and tonic discharges (Donnelly, 1999).

Patch clamp recordings of rat isolated chemosensory neurons indicate that they present both transient and persistent TTX-sensitive Na^+^ currents. Conversely, non-chemosensory PG neurons persistent Na^+^ current is TTX-insensitive (Cummins et al., 2002). Molecular biology determinations indicate that both TTX-resistant (Na_V_ 1.8, Na_V_ 1.9) and TTX-sensitive (Na_V_ 1.1, Na_V_ 1.6, Na_V_ 1.7) Na^+^ channel isoforms are expressed in the rat PG (Cummins et al., 2002). Rat chemosensory neurons express several K^+^ channels that underlie delayed rectifier currents (K_V_ 1.2, K_V_ 1.5, K_V_ 1.6, K_V_ 2.1), fast transient and inactivating currents (K_V_ 1.4, K_V_ 4.3), M-currents (KCNQ2, KCNQ3, KCNQ5), Ca^2+^-dependent (KCa1.1) currents (Andrews and Kunze, 2001; Buniel et al., 2008), and also, hyperpolarization-activated cyclic nucleotide-gated (HCN2, HCN4) channels (Buniel et al., 2008). However, a large variation in channel expression occurs within the chemosensory neurons reflected in different whole cell currents (Andrews and Kunze, 2001). Thus, although PG neurons appear as a homogeneous population, chemosensory neurons present subtle differences that could have different physiological and regulatory meaning.

Mechanosensory neurons appear to comprise a population of large, fast conducting neurons that present fast APs with short hyperpolarizations, generated by a TTX sensitive Na^+^-current and a TEA-sensitive K^+^-current, respectively. On the other hand, chemosensory neurons present APs of longer duration and long lasting hyperpolarization, resulting from the presence of Ca^2+^-currents and a Ca^2+^-dependent K^+^-current, respectively, as well as other K^+^-currents.

### Responses to neurotransmitters

Many transmitter molecules have been indicated to participate in the generation and/or modulation of carotid chemosensory activity (González et al., 1994; Iturriaga and Alcayaga, 2004; Nurse and Piskuric, 2013). However, the postsynaptic effects have been slowly unraveling in the last decades (Iturriaga and Alcayaga, 2004; Nurse and Piskuric, 2013).

Cat and rabbit PG neurons projecting through the CSN increase their AP discharge frequency in response to acetylcholine (ACh), effect blocked by nicotinic ACh receptor (nAChR) antagonists (Alcayaga et al., 1998; Soto et al., 2010). In the cat, the activation of ACh muscarinic receptors (mAChRs) appears to be devoid of effects (Alcayaga et al., 1998) but in rabbits antagonizing mAChRs increased the magnitude of the ACh-induced responses, suggesting an inhibitory action for these receptors (Soto et al., 2010). Most (96%) identified cat chemosensory PG neurons depolarized and generated APs when ACh is applied to the soma while none of the barosensory ones responded (Varas et al., 2003), although activation of barosensory afferents by ACh and nicotine has been reported (Diamond, 1955). Nevertheless, activation of afferents with ACh and nicotine was obtained in the pressurized carotid bifurcation and vascular effects cannot be ruled out.

In cultured PG neurons ACh and nicotine induces depolarization (Zhong and Nurse, 1997; Varas et al., 2000) which is blocked by nAChR antagonists (Zhong and Nurse, 1997; Shirahata et al., 2000; Varas et al., 2006; Alcayaga et al., 2007). Agonist and antagonist sensibility indicate the presence of both α7 and α4β 4 (Shirahata et al., 2000; Varas et al., 2006) or α4β 2 nAChRs (Shirahata et al., 2000), in concordance with immunohistochemical evidence (Shirahata et al., 1998, 2000).

In a reconstituted system, containing rat NPJc neurons and CB cells, the basal neuronal activity as well as hypoxia induced increases in neuronal activity are partially blocked by nAChRs antagonists (Zhong et al., 1997; Nurse and Zhang, 1999, 2001; Zhang et al., 2000; Zhang and Nurse, 2004) and increased by an acetylcholinesterase inhibitor (Nurse and Zhang, 1999, 2001). These evidences indicate that in this preparation the basal and hypoxia–and hypercapnia-induced activity in NPJc neurons can be partly blocked by nAChRs antagonists.

The rabbit and cat PG neurons projecting through the CSN increase their spiking activity in response to ATP in a dose dependent manner (Alcayaga et al., 2000; Soto et al., 2010). These responses are blocked by P2X but not by P2Y antagonism (Alcayaga et al., 2000). Most (93%) identified chemosensory cat PG neurons respond to application of ATP to the perikarya with depolarization and spike trains while only 40% of barosensory neurons responded (Varas et al., 2003).

Whole cell recordings of cultured cat PG neurons show that ATP induces a dose dependent depolarization that increased the discharge frequency and a sustained inward current at a holding potential near the resting Vm (−60 mV) (Alcayaga et al., 2007). These responses are mimicked by α,β-methylene ATP and blocked by suramin, suggesting the involvement of P2X2,3 receptors in the generation of these responses (Alcayaga et al., 2007). In an *in vitro* preparation containing NPJc neurons and CB cells obtained from Sprague-Dawley rats, that respond as a reconstituted arterial chemoreceptor (Zhong et al., 1997), hypoxia- and hypercapnia-induced responses recorded from PG neurons are partially blocked by reduced extracellular Ca^2+^/Mg^2+^ ratio and suramin, a nucleotide receptor blocker. These data indicate a synaptic, ATP-mediated activation of PG neurons (Zhang et al., 2000; Nurse and Zhang, 2001; Prasad et al., 2001; Zhang and Nurse, 2004). The presence of P2X2 and P2X3 subunits has been demonstrated in single cell RT-PCR of responding neurons in this preparation and by confocal immunofluorescence of rat NPJc neurons and neuronal terminals in the CB (Prasad et al., 2001). Moreover, mice lacking P2X2 and P2X3 subunits present reduced chemosensory afferent discharges and ventilatory responses (Rong et al., 2003). Nevertheless, the presence of P2X1 subunits has been reported in PG homogenates and other P2X subunits (i.e., P2X5, P2X7) may participate in ATP-induced intracellular Ca^2+^ increases in PG neurons (Nunes et al., 2012).

Recordings of identified cat chemosensory neurons (Varas et al., 2003) and neurons in a reconstituted system (Zhang et al., 2000; Nurse and Zhang, 2001) show that the responses induced by chemoreceptor stimuli are partially block by either nAChRs or nucleotide receptors blockade, and completely abolished by the joint administration of the blockers (Zhang et al., 2000; Nurse and Zhang, 2001; Varas et al., 2003). These results show that at least in these preparations chemosensory afferent activity depends almost exclusively on the synaptic communication mediated by ACh and ATP. Nevertheless, the afferent activity recorded from the CSN *in situ* and CB superfused *in vitro* indicates that responses induced by ACh and ATP are blocked by their respective blockers while ventilatory (Reyes et al., 2007b) and afferent activity remain largely unaffected (Reyes et al., 2007a,b) or only partially inhibited (Fitzgerald et al., 1997).

**Figure 2:**
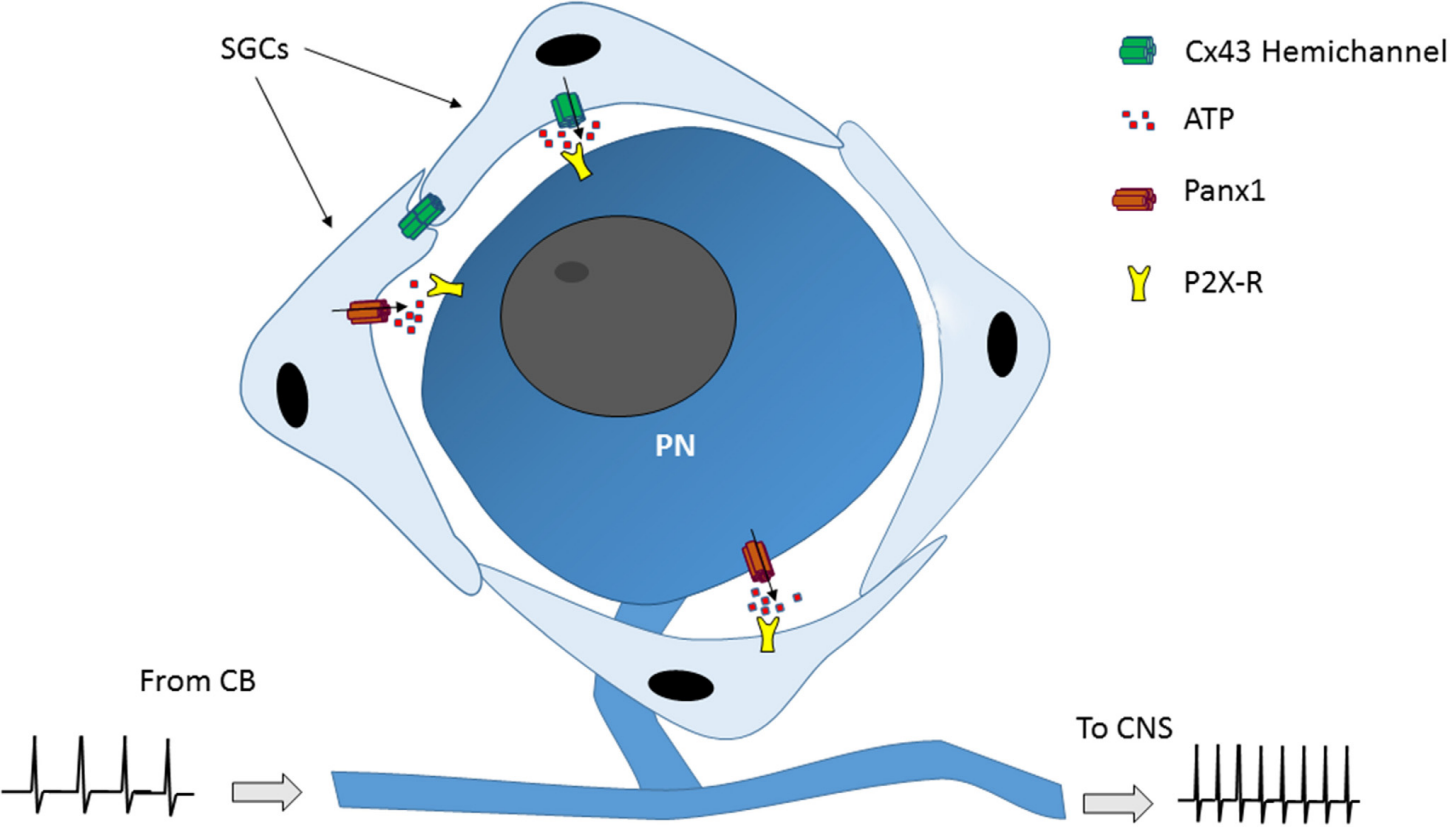
**Model of how satellite glial cells (SGCs) could modulate the petrosal neuronal activity**. Connexin43 (Cx43) hemichannels and/or pannexin 1 (Panx1) channels present in SGCs when open allows the release of neurotransmitters to the extracellular space. Then, the neurotransmitters (i.e., ATP) activate receptors (i.e., P2X-R) in petrosal ganglion neurons (PN), which in turn induce their depolarization, which in turn may modulate the information from the carotid body (CB) to the CNS.

Dopamine is another neurotransmitter involved in the CB-petrosal neuron communication. The presence of D1- and D2-dopamine receptor mRNA in rat and cat PG (Schamel and Verna, 1993; Gauda et al., 1994; Bairam et al., 1998) and the enzyme tyrosine hydroxylase (TH) (Katz et al., 1983) have been demonstrated. The content of TH and the number of neurons expressing TH is increased after neuronal depolarization with KCl (40 mM) or veratridine (neurotoxin abolishing inactivation of Na^+^ channels) (Hertzberg et al., 1995), suggesting that the activity of petrosal neurons is a modulator of the TH expression in this neurons. Moreover, about 25% of petrosal neurons release dopamine in response to depolarizing signals *in vitro* (Iturriaga et al., 2003). Dopamine is released by CB cells in response to hypoxia (Hellström et al., 1976; Iturriaga et al., 1996, 2009; Iturriaga and Alcayaga, 1998) where it inhibits afferent activity (Zapata, 1975). However, when dopamine is applied in repetitive doses in short intervals the response becomes biphasic, observing an initial inhibition followed by an activation of the afferent discharge (Zapata, 1975). Similarly, low doses (<10 μg) of dopamine applied directly to the cat PG *in vitro* enhance the responses to ACh (Alcayaga et al., 1999), while higher doses (>200 μg) inhibit the responses to both ACh and ATP (Alcayaga et al., 1999, 2003a) (Figure 2). The inhibition induced by dopamine is blocked by spiroperone (Alcayaga et al., 1999), a D2 antagonist. Recently, it has been reported that sensory neurons projecting through the vagus nerve increase their activity in response to a media without Ca^2+^ and Mg^2+^, increase that is blocked by dopamine (Retamal et al., 2014). All these data support the notion that dopamine is an inhibitory neurotransmitter for neurons in the PG and more specifically in those projecting to the CB. However, DA application to the rabbit PG produces a dose dependent increase in the neuronal discharge (Iturriaga et al., 2009), suggesting that the actions of a determined transmitter may be species specific.

The aforementioned data indicate that PG neurons that project to the CB are excited, at least, by ACh and ATP and those responses can also be modulated by, dopamine. However, the lack of complete elimination of responses to hypoxia *in situ* indicate that other transmitter molecules may also be involved in the generation of the afferent activity.

## Have the petrosal ganglia all the necessary elements for information processing?

As mentioned before, PG neurons express several different types of receptors -including ionotropic and metabotropic- in their somas. Rabbit's but not rat's petrosal neurons change their response to neurotransmitters as a consequence of normobaric chronic hypoxia (Iturriaga and Alcayaga, 2007; Icekson et al., 2013). However, in both preparations the CB chemosensory response to hypoxia was enhanced (Barnard et al., 1985; Iturriaga, 2013), suggesting that changes in the PG induced by changes in the oxygen level are not completely dependent on the activity of the CB. On the other hand, partial or complete elimination of the PG afferences have been used to treat some pathologies.

An increasing body of evidence shows that there is information processing into sensory ganglia related to the appearance and/or maintenance of chronic pain. Thus, satellite glial cells (SGCs) of trigeminal and dorsal root ganglion are activated in response to neuronal damage and/or to inflammatory process. This activation -correlated with an increased sensory neurons activity- (Blum et al., 2014; Song et al., 2014; Warwick et al., 2014) was importantly reduced by the intraganglionar application of connexin (Cx)-channel blockers (Dublin and Hanani, 2007; Huang et al., 2010; Hanani, 2012). It has been postulated that the cross-talk between neurons and SGCs is mediated by ATP release (through an undetermined pathway) from those cells (Suadicani et al., 2010). Cx- hemichannels and pannexin (Panx)-channels have been reported to be involved in autocrine and paracrine communication in several systems, including the central nervous system (Sáez et al., 2003; Orellana et al., 2013). Petrosal neurons and their surrounding SGCs express at least Cx43 (Chen et al., 1985; Retamal et al., 2014) and may also express Panx1, which was found in the whole NPJc (Retamal et al., 2014). Thus, if this type of paracrine communication is present in petrosal ganglia, the opening of hemichannels in petrosal neurons and/or in SGCs could lead to an increase in the petrosal neurons activity (Figure 2), which in turn could induce for example diseases related to cardiovascular disorders. Thus, for example, CSN denervation partially corrected the sympathetic and respiratory variables in rabbits with experimental congestive heart failure (Marcus et al., 2014).

## Future directions

Since there are no works showing information processing in the PG, it is necessary to perform experiments in order to explore the possibility that SGCs and petrosal neurons communicate. This could be due to the release of neuro- and/or glio-transmitters through Cx hemichannels or Panx channels, as suggested by Retamal et al. (2014). Therefore, research on the communication of petrosal neurons and SGCs may answer questions concerning the oxygen or arterial pressure information processing such as; is there any effect of Cx or Panx channel blockers in the oxygen or arterial pressure information processing? Do Cx or Panx knockout animals present differences in that processing? Are SGCs important for the maintenance of normal neuronal activity? Since the expression of receptors and channels in petrosal neurons is modified by hypoxia and others stimulus, it could be interesting to assay if those modifications also modify the information from the CB to the CNS. As discussed, PG have all the necessary elements for information processing, hence it can modulate the information conveyed from the CB to the CNS, thus future study could focus in this putative modulation.

### Conflict of interest statement

The authors declare that the research was conducted in the absence of any commercial or financial relationships that could be construed as a potential conflict of interest.
